# Accurate detection of somatic single-nucleotide variants from bulk RNA-seq data using RNA-MosaicHunter

**DOI:** 10.1093/nar/gkaf1450

**Published:** 2026-01-08

**Authors:** August Yue Huang, Yuchen Cheng, Jayoung Ku, Boxun Zhao, Junseok Park, Dachan Kim, Jaejoon Choi, Eunjung Alice Lee

**Affiliations:** Division of Genetics and Genomics and Manton Center for Orphan Disease Research, Boston Children’s Hospital, Boston, MA 02115, United States; Department of Pediatrics, Harvard Medical School, Boston, MA 02115, United States; Broad Institute of MIT and Harvard, Cambridge, MA 02142, United States; Division of Genetics and Genomics and Manton Center for Orphan Disease Research, Boston Children’s Hospital, Boston, MA 02115, United States; Department of Biomedical Informatics, Harvard Medical School, Boston, MA 02115, United States; Division of Genetics and Genomics and Manton Center for Orphan Disease Research, Boston Children’s Hospital, Boston, MA 02115, United States; Department of Pediatrics, Harvard Medical School, Boston, MA 02115, United States; Broad Institute of MIT and Harvard, Cambridge, MA 02142, United States; Division of Genetics and Genomics and Manton Center for Orphan Disease Research, Boston Children’s Hospital, Boston, MA 02115, United States; Department of Pediatrics, Harvard Medical School, Boston, MA 02115, United States; Broad Institute of MIT and Harvard, Cambridge, MA 02142, United States; Division of Genetics and Genomics and Manton Center for Orphan Disease Research, Boston Children’s Hospital, Boston, MA 02115, United States; Department of Pediatrics, Harvard Medical School, Boston, MA 02115, United States; Division of Genetics and Genomics and Manton Center for Orphan Disease Research, Boston Children’s Hospital, Boston, MA 02115, United States; Department of Pediatrics, Harvard Medical School, Boston, MA 02115, United States; Department of Otorhinolaryngology, Severance Hospital, Yonsei University Health System, Yonsei University College of Medicine, Seoul 03722, South Korea; Division of Genetics and Genomics and Manton Center for Orphan Disease Research, Boston Children’s Hospital, Boston, MA 02115, United States; Department of Pediatrics, Harvard Medical School, Boston, MA 02115, United States; Broad Institute of MIT and Harvard, Cambridge, MA 02142, United States; Division of Genetics and Genomics and Manton Center for Orphan Disease Research, Boston Children’s Hospital, Boston, MA 02115, United States; Department of Pediatrics, Harvard Medical School, Boston, MA 02115, United States; Broad Institute of MIT and Harvard, Cambridge, MA 02142, United States

## Abstract

Somatic variants are increasingly recognized as contributors to diverse non-cancer, developmental, and aging-related disorders. However, most tools for detecting somatic single-nucleotide variants (sSNVs) were designed for DNA sequencing and primarily tailored to cancer datasets, leaving a critical gap in harnessing the rich potential of RNA-seq for sSNV identification, particularly in non-cancer tissues with low mutation rates. Here, we introduce RNA-MosaicHunter, a novel bioinformatic tool for accurate sSNV detection from bulk RNA-seq. In two benchmarking datasets, it demonstrated high precision (94.7% in TCGA and 99.3% in a cell-line mixture) with sensitivities of 53.4% and 38.9%, respectively, in the default mode that maximizes precision. We then applied RNA-MosaicHunter to profile 827 RNA-seq samples in three tissue types from the Genotype Tissue Expression project (GTEx), where it outperformed previous methods in capturing mutational characteristics associated with normal aging. We further utilized RNA-MosaicHunter to analyze RNA-seq data from 382 Alzheimer’s disease (AD) brain samples and 480 age-matched controls and revealed a significantly higher burden of sSNVs in AD cerebral cortex, suggesting the potential contribution of sSNVs to AD pathogenesis. RNA-MosaicHunter enables accurate profiling and characterization of sSNVs from RNA-seq data, advancing the understanding of the role of somatic variants across diverse tissues and diseases.

## Graphical Abstract

**Graphical Abstract:**
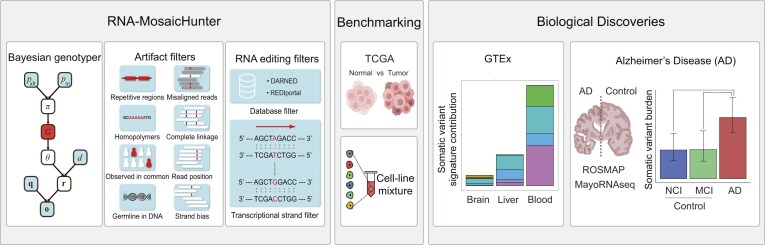

## Introduction

Somatic variants arise from inevitable errors in DNA replication and exposures to exogenous and endogenous mutagenesis factors [1, 2]. Clonal somatic variants, which are shared by a subset of cells, typically arise early in embryogenesis or undergo clonal expansion driven by natural selection; this enables them to reach a high allele fraction, making them detectable from sequencing of bulk tissue samples [3]. Increasing evidence supports that clonal somatic variants are closely associated with human diseases. It has long been known that gain-of-function somatic variants in oncogenes and loss-of-function variants in tumor suppressor genes contribute to cancer development [4, 5]. More recently, the pathogenic role of somatic variants has been revealed in more and more non-cancerous diseases, including Mendelian monogenic diseases such as Proteus syndrome [6] and congenital malformations [7, 8], as well as non-Mendelian complex diseases such as congenital heart disease [9], autism spectrum disorders [10], and neurodegenerative diseases [11]. Additionally, somatic variants accumulate in normal tissues during early development and aging across different types of tissues [12–16]. For instance, somatic variants in hematopoietic cells induce clonal hematopoiesis [13], which has been associated with increased risks of hematological neoplasms [17] and cardiovascular disease [18].

With the rapid development of next-generation sequencing techniques in the last few decades, the identification of somatic variants from sequencing data has become available. Due to the limitations of sequencing technologies, including base-calling and alignment errors [19], many computational tools have been developed for somatic variant calling from DNA-seq, first designed for cancer samples and then adapted for non-cancer samples [20–24]. Compared to DNA-seq, bulk and single-cell RNA-seq have more datasets available generated for transcriptome profiling, demonstrating a huge potential for somatic variant detection. However, RNA-seq data have unique features that need to be addressed for somatic variant calling. First, the exon-intron structure in mRNA requires the spliced alignment of RNA-seq reads onto the human reference genome, which increases the chance of alignment errors when the overhang sequence is relatively short [25]. Second, the widespread adenosine-to-inosine (A>I) RNA-editing sites across the human genome [26] are indistinguishable from A>G somatic variants in RNA-seq data, because sequencers recognize inosine as guanine (G). Third, the allele-specific expression [27], a phenomenon in which the paternal and maternal alleles have different expression levels, is observed in many autosomal and X chromosome genes, leading to deviated allele fraction estimation in RNA-seq data. Lastly, RNA-seq coverage is more variable across the genome and between samples than DNA-seq coverage, primarily due to the wide range of expression levels among genes and their isoforms.

Currently, most single-cell RNA-seq datasets are based on the 10X Genomics platform, which only sequences the 5′ or 3′ end of mRNA molecules, thus limiting the capability for variant calling across the entire protein-coding region. In contrast, bulk RNA-seq provides better transcriptome-wide coverage for expressed genes. Early efforts to detect somatic variants from bulk RNA-seq data primarily used cancer datasets, which paved the way for demonstrating its feasibility [28–31]. More recently, a few methods have been developed for non-cancer bulk RNA-seq, including RNA-MuTect [32] and RnaMosaicMutationFinder [33]. RNA-MuTect utilizes MuTect [23], originally designed for detecting cancer somatic variants from DNA-seq data as its backbone, and integrates a series of RNA-specific filters, such as an RNA-seq-derived panel of normals and RNA editing databases. RnaMosaicMutationFinder incorporates a random forest model with parameters trained on lymphocytic leukemia samples [33]. Both methods are based on models tailored to cancer datasets, which may limit their performance on non-cancer datasets, largely due to the substantially lower occurrence rate and variant allele fractions (VAFs) for somatic variants in non-cancer samples.

To address the limitations of the previous tools, we introduce RNA-MosaicHunter, a novel somatic variant caller for bulk RNA-seq data. Building on MosaicHunter [22, 34], specifically developed for somatic variant calling from non-cancer DNA-seq data, RNA-MosaicHunter integrates a Bayesian genotyper and a series of empirical filters to distinguish real somatic variants from sequencing artifacts and RNA-editing sites. We benchmarked the performance of RNA-MosaicHunter on cancer and normal tissue datasets and demonstrated that it outperforms previous tools in profiling mutational burdens and signatures. We further applied RNA-MosaicHunter to cohorts of Alzheimer’s disease (AD) patients and matched controls, revealing an increasing burden of somatic variants in the AD cerebral cortex, which highlights their potential role in AD pathogenesis.

## Materials and methods

### Design of RNA-MosaicHunter

**Figure 1.**
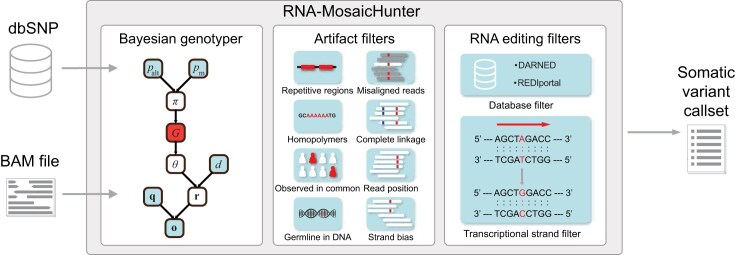
Overview of the RNA-MosaicHunter for detecting clonal somatic variants from RNA-seq data. Candidate sSNVs were captured by a Bayesian genotyper, followed by a series of artifact and RNA editing filters. Using the aligned BAM file as input, the Bayesian genotyper estimated the posterior probabilities of mosaic or germline genotypes by incorporating base-calling errors, random sampling variations, and population allele frequencies reported in dbSNP. Additionally, artifact filters were incorporated to eliminate false positives resulting from systematic base-calling and read alignment errors, as well as other genomic variants like structural variations and indels. We further designed specific filters to remove RNA-editing sites from the somatic variant call set by considering public RNA editing databases as well as substitution type and gene-transcribed information of each candidate.

Here, we introduce RNA-MosaicHunter, a new bioinformatic tool designed to identify somatic single-nucleotide variants (sSNVs) from bulk RNA-seq data. Derived from DNA-seq-based MosaicHunter [22, 34], RNA-MosaicHunter consists of two major components: a Bayesian genotyper to distinguish real variants from base-calling errors, followed by a series of filters to remove artifacts introduced from various sources and RNA-editing sites (Fig. 1).

In the Bayesian genotyper, *G* denotes the genotype state, π denotes the prior probability of each genotype inferred from the population variant allele frequency *p*_*alt*_and default somatic variant rate *p_m_. d, q*, and *o* denote the depth, base qualities, and bases for calculating genotype likelihoods from the observed sequencing data, respectively. Since the VAF in RNA-seq data can be affected by allele-specific expression or copy number alterations, we considered the posterior probability of both germline heterozygous genotype and somatic mosaic genotype in our list of variant candidates for subsequent error filters and further distinguished somatic variants from germline variants by using the genotyping results from matched whole-genome or whole-exome sequencing data obtained from the same individual. RNA-MosaicHunter can also be run on RNA-seq samples without matched DNA-seq data. In this case, we recommend that users utilize population polymorphism databases such as dbSNP [35, 36] and gnomAD [37] to filter out germline variants. As germline variants generally have a median VAF around 0.5, whereas somatic variants usually show lower VAFs because they are present in only a subset of cells, users can further enrich for true somatic variants and reduce potential germline contamination by applying a maximum RNA-seq VAF threshold of 0.5 or lower when matched DNA-seq data are unavailable.

RNA-MosaicHunter also incorporated error filters to exclude (i) somatic candidates with <5% VAF or <5 variant-supporting reads; (ii) somatic candidates in repetitive and homopolymer regions; (iii) somatic candidates with a significant bias in read strand, mapping quality, or within-read position between the reference and variant alleles; (iv) somatic candidates that show complete linkage to adjacent somatic candidates on the same read or read pairs, which is more likely to be caused by alignment errors; and (v) somatic candidates that are supported by fewer than 50% of “high-quality” reads among all reads covering that position, in which “high-quality” reads are defined as those whose alignment has been confirmed by a second aligner BLAT [38] and those where the candidate was not located near the start, end, or spliced junctions of the read. To exclude A>I(G) RNA-editing sites, we removed all previously identified editing sites reported in DARNED [39] and REDIportal [40]. Additionally, we removed all A>G candidates on the transcribed strands and T>C candidates on the untranscribed strands of any genes to account for potential RNA-editing sites that have not yet been reported. We also offered an option to fully remove A>G/T>C variants for higher precision. The source code and default configuration file of RNA-MosaicHunter are publicly available at https://github.com/AugustHuang/RNA-MosaicHunter, and they support users to customize parameters for the Bayesian genotyper and empirical error filters.

RNA-MosaicHunter can complete somatic variant calling for a typical RNA-seq dataset (with ∼50 million 151-bp paired-end reads) in 3–5 hours using one CPU core and 32 GB of memory. It also supports analyzing each chromosome separately, which can significantly reduce the memory requirement and enable parallelization to utilize multiple cores for a given RNA-seq dataset.

### Somatic variant calling from RNA-seq data

Each downloaded RNA-seq BAM file was first converted back to the FASTQ format by Picard (v1.138) and then aligned to the GRCh37 human reference genome by STAR (v2.5.0a) [41] in the two-pass mode, where the reference gene annotation (Gencode version 19) was used in the first pass, and then a sample-specific annotation generated from the first pass was used in the second pass. The aligned reads were processed by Picard (v1.138) to remove duplicates, followed by SplitNCigarReads, indel realignment, and base quality recalibration of GATK (v3.6) [42]. Reads that were improperly paired or with ambiguous alignment were removed, and only genomic positions covered by 10 or more reads were subject to RNA-MosaicHunter.

We further excluded non-exonic candidates and candidates that are present in the polymorphism databases of the general human population, including dbSNP [35], the 1000 Genomes Project [43], the Exome Sequencing Project [44], and the Exome Aggregation Consortium [45].

### Analysis of The Cancer Genome Atlas (TCGA) dataset

RNA-seq and whole-exome sequencing (exome-seq) data of 19 esophageal carcinoma samples as well as exome-seq data of their matched normal samples were downloaded from the TCGA Research Network (Supplementary Table S1) [46]. Somatic variants were called from the tumor RNA-seq samples using RNA-MosaicHunter with the default pipeline and parameters (Supplementary Table S2). We excluded somatic candidates shared across multiple tumor samples, as they were likely common sequencing artifacts, although this approach may inadvertently remove a small number of recurrent true cancer driver variants. Somatic variant calls created by the Broad Institute through the comparison of tumor and matched normal exome-seq pairs using MuTect [23] were also downloaded. We estimated the sensitivity and precision of our model to evaluate the performance compared to the MuTect call set. The sensitivity was calculated as the percentage of somatic variants reported by MuTect that were recaptured by RNA-MosaicHunter, within the genomic regions covered by 10 or more reads in RNA-seq. The precision was estimated as the percentage of somatic variants called by RNA-MosaicHunter that had also been called by MuTect from tumor DNA-seq. Somatic variants missed by MuTect but exhibiting a >2% VAF in tumor DNA-seq and absent in control DNA-seq were additionally considered as true somatic variants. We further assessed the performance of RNA-MosaicHunter with the removal of each single filter and without any filters by modifying the configuration file.

### Cell line mixture preparation and RNA sequencing

Six human lymphoblastoid cell lines (GM12878, GM18620, GM18865, GM19141, GM20126, and GM20904) were kindly provided by Kathleen Burns’s lab at the Dana Farber Cancer Institute. All cell lines were maintained in RPMI 1640 medium (Corning, Cat# 10-040-CV) supplemented with 2 mM l-glutamine and 15% fetal bovine serum (Gibco). Two biological replicates of the cell mixture were independently prepared, each consisting of 5 million viable cells as determined by a trypan blue exclusion assay. For each replicate, the six cell lines were mixed based on cell counts in the following proportions: GM12878 (45%), GM18620 (20%), GM18865 (10%), GM19141 (10%), GM20126 (10%), and GM20904 (5%). Total RNA was isolated using the Quick-RNA Miniprep kit (Zymo Research, Cat# R1055) according to the manufacturer’s protocol. For each replicate, a stranded mRNA-seq library was constructed using the Illumina Stranded mRNA Prep kit and sequenced on an Illumina NovaSeqX platform to generate ~160 million 151-bp paired-end reads per library.

### Analysis of the cell-line mixture dataset

Germline variants of the six cell lines, called from whole-genome sequencing (WGS) data, were downloaded from the 1000 Genomes Project [47] in the VCF format. Variants were merged by GATK (v4.6.1) [48] and then filtered by bcftools (v1.21) based on read depth >20 in all six lines to obtain high-confidence germline variant calls [49]. Expected DNA VAFs were calculated based on genotype and mixing proportion of the lines and were further restricted to regions with RNA-seq depth >10 and to exonic variants defined by ANNOVAR [50] and RefSeq [51]. These variants were considered the true set.

Somatic variants from the two cell-line mixture replicates were detected using RNA-MosaicHunter, with the same RefSeq-based exonic filter applied as for the true set (Supplementary Table S3). Unlike the standard pipeline, we did not apply the polymorphism database filter to the cell-line mixture call set, because these germline variants used to simulate somatic variants are almost all common single-nucleotide polymorphisms (SNPs) cataloged in such databases.

As in the TCGA benchmarking, sensitivity and precision were evaluated using the full set of filters, with individual filters removed, or with all filters removed. For both the complete call set and bins stratified by expected DNA VAF, RNA depth, and RNA VAF, true positives were defined as variants found in both the RNA-MosaicHunter call set and the true set, whereas false negatives were defined as variants in the true set that were absent from the RNA-MosaicHunter calls. False positives were defined as RNA-MosaicHunter-called variants absent from the DNA-based true set in both the full set and within each bin, except for DNA VAF bins, where all false positives were considered since they cannot be assigned to specific DNA VAF bins.

### Analysis of the Genotype-Tissue Expression dataset

Metadata for the Genotype-Tissue Expression (GTEx) project were downloaded from dbGaP accession number phs000424.v9.p2 on 02 September 2024 (https://gtexportal.org/home/aboutAdultGtex). We extracted all RNA-seq samples from the brain cortex, cerebellum, hippocampus, liver, and whole blood for which WGS data from the same individual are available. After removing duplicated samples, we curated a final sample list consisting of 423 individuals with WGS and 827 RNA-seq datasets (Supplementary Table S4). BAM files for WGS and fastq files for RNA-seq were downloaded from Google Cloud using SRA-Toolkit (v3.0.10). Clonal somatic variants in GTEx data were called using RNA-MosaicHunter with the default pipeline and parameters. We excluded somatic candidates shared by multiple individuals, as they were likely to be common sequencing artifacts. Consistent with previous studies on the GTEx dataset, we observed a substantial contribution of G>T candidates on the gene-transcribed strand in our call lists, a pattern most likely attributable to 8-oxo-guanine DNA oxidation artifacts in GTEx samples [32]. Such artifacts have been frequently reported in somatic variant studies using post-mortem samples [52–54]. Therefore, we further removed G>T candidates detected on the transcribed strand and C>A candidates detected on the untranscribed strand of any genes. Somatic variants detected from RNA-seq by RNA-MosaicHunter were summarized in Supplementary Table S5.

The somatic variant call sets for GTEx samples, generated by RNA-Mutect [32] and RnaMosaicMutationFinder [33], were extracted accordingly from their supplementary tables for the relevant tissue types. To estimate the expected number of clonal somatic variants in RNA-seq powered regions of normal brain tissues, we extracted a list of somatic variants identified from deep (∼250×) WGS data of 15 normal brain samples [55]. We then calculated the average number of somatic variants within the genomic regions covered by 10 or more reads in GTEx brain RNA-seq datasets.

For mutational signature analysis, we identified the top 10 COSMIC signatures with the highest contributions across three TCGA cancer types (glioblastoma, liver cancer, and acute myeloid leukemia) based on signature decomposition. We chose these three cancer types because we analyzed their corresponding normal tissue types in GTEx (brain, liver, and blood). We then performed signature refitting for the variants detected by RNA-MosaicHunter in GTEx samples against these 10 signatures. The mutational spectrum was normalized by considering trinucleotide frequencies in RNA-seq powered regions before signature decomposition. MAFtools (v2.12.0) [56] was used for gene-level annotations for detected variants. Clonal hematopoiesis of indeterminate potential (CHIP) variants were annotated based on previously reported gene sets [57, 58].

### Analysis of the Alzheimer’s disease datasets

Two large-scale AD cohorts, ROSMAP [59] and MayoRNAseq [60], were included in our somatic variant analyses. The ROSMAP study integrates two longitudinal aging studies, namely the Religious Order Study (ROS) and the Memory and Aging Project (MAP), conducted by the Rush Alzheimer’s Disease Center. Participants in these studies underwent comprehensive cognitive and neuroimaging assessments and detailed neuropathological evaluations during autopsy. The MayoRNAseq study involved thorough clinical phenotyping and multi-omic profiling of 300 samples provided by the Mayo Clinic Brain Bank and the Banner Sun Health Research Institute. AD diagnosis was established based on a consensus review of all postmortem data by neurologists specializing in dementia and neurodegenerative disorders. Sample information was summarized in Supplementary Table S6.

The BAM files of RNA-seq and VCF files containing germline variant calls from matched WGS data, generated by the ROSMAP and MayoRNAseq studies, were obtained from the AMP-AD Knowledge Portal. These files were accompanied by comprehensive demographic and clinical data for each sample. Supplementary Table S7 summarized all the bulk brain RNA-seq samples analyzed for somatic variant calling. The ROSMAP dataset includes prefrontal cortex (PFC) samples from 225 AD patients and 337 age-matched controls with no or mild cognitive impairment, collected through the ROSMAP project. The MayoRNAseq dataset comprises temporal cortex and cerebellum samples from 92 AD patients and 82 age-matched controls, with most participants having RNA-seq data from both brain regions. RNA-MosaicHunter with default parameters was utilized to identify somatic variants from each RNA-seq sample of ROSMAP and MayoRNAseq. Somatic candidates shared by more than two individuals in ROSMAP or MayoRNAseq were excluded. For added stringency in the context of disease-related studies, we further excluded all A>G and T>C candidates to achieve higher precision. We further annotated all somatic candidates using ANNOVAR [50] and classified a candidate as deleterious if it was annotated as splicing, stop-gain, or stop-loss, or if it was a missense variant predicted to be “deleterious” by either PolyPhen-2 [61] or SIFT [62].

To estimate the proportion of neurons and other brain cell types in each RNA-seq sample, we applied CIBERSORT (v1.05) [63] to deconvolute the cell-type composition by using the cell-type-specific expression reference for different neuronal and glial types (excitatory and inhibitory neuronal subtypes in the cortex, cerebellar granule cells, Purkinje cells, endothelial cells, pericytes, astrocytes, oligodendrocytes and their precursor cells, and microglia), generated from a large-scale brain single-cell RNA-seq dataset [64]. We summed the estimated proportion of all subtypes of excitatory and inhibitory neurons to calculate the overall neuronal proportion for each sample.

Somatic variant density in each clinical group was calculated by counting the total number of somatic variants and dividing it by the total size of powered regions with ≥10× RNA-seq coverage, and the odds ratio and the two-sample *Z*-test of proportion were used to test whether the AD group had a higher burden of somatic variants than the control group. For the linear regression analysis, the count of somatic variants in each sample was modeled as a continuous outcome, whereas clinical status and other covariates of interest (e.g. age, sex, sequencing depth, post-mortem interval, and neuronal proportion) were modeled as independent variables. In regression analysis, we only considered donors with ages <90, because all the donors with age 90 or higher were labeled as “90+” in the demographic tables of the ROSMAP and MayoRNAseq studies.

Functional enrichment analysis of Gene Ontology (GO) terms was performed using GOseq (v1.34.1) [65]. Exonic somatic variants identified from the RNA-seq of AD patients or normal controls were used as the input, and Wallenius’ noncentral hypergeometric distribution was used to test the enrichment, with a probability weighting function to control for potential gene length bias. Only GO terms with three or more hits and an initial overrepresentation *P*-value <.01 were considered. GO terms with >1000 genes were excluded. The *P*-value was adjusted by Hommel’s method for the correction of multiple hypothesis testing.

## Results

### RNA-MosaicHunter demonstrates high sensitivity for somatic variant calling in cancer datasets

**Figure 2.**
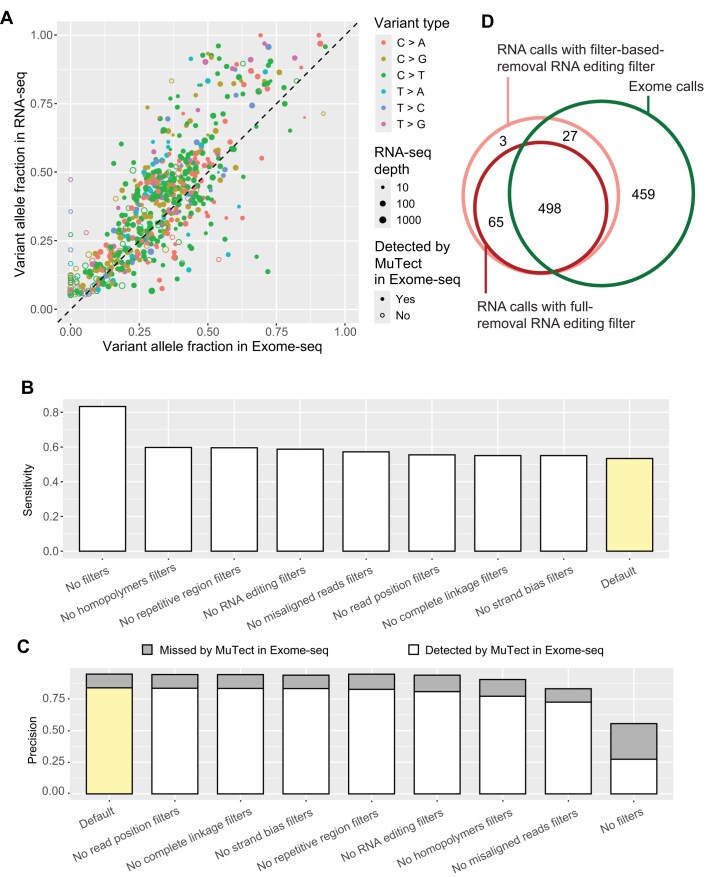
Benchmarking the performance of RNA-MosaicHunter using the TCGA cancer data. (**A**) Comparison of variant allele fraction of somatic variants detected by RNA-MosaicHunter from RNA-seq versus those detected by MuTect in the matched exome-seq data in 19 esophageal carcinoma samples. Five hundred twenty-five of 626 sSNVs identified by RNA-MosaicHunter were confirmed by MuTect in the matched exome-seq data (filled circles). RNA-MosaicHunter recaptured 68 sSNVs present in exome-seq but missed by MuTect (open circles). Sensitivity (**B**) and precision (**C**) for RNA-MosaicHunter in default mode (yellow) or after removing specific filters (white). In default mode, RNA-MosaicHunter achieves 53.4% sensitivity and 94.7% precision; precision increases slightly to 94.9% when T>C/A>G candidates, which are vulnerable to RNA editing, are excluded. The gray bar in the precision plot indicates variants detected by RNA-MosaicHunter that are present in exome-seq with >2% VAF but missed by MuTect. (**D**) Venn diagram of somatic variants called by RNA-MosaicHunter in the default RNA-editing removal mode from RNA-seq (pink), RNA-MosaicHunter with full RNA-editing removal (red), and MuTect from exome-seq (green). Numbers of unique and shared variants are labeled in the corresponding circles.

To benchmark the performance of RNA-MosaicHunter, we first utilized data of 19 esophageal carcinoma samples obtained from the TCGA Network (Supplementary Tables S1 and S2) [46]. We used the somatic variant call set generated by MuTect [23] from exome-seq of tumor–normal pairs as the true reference set and then applied RNA-MosaicHunter to the RNA-seq data from the same tumor samples. By using the default parameters, RNA-MosaicHunter identified 626 sSNVs from the tumor RNA-seq data, and 525 of them were also called by MuTect from matched exome-seq data, confirming the accuracy of RNA-MosaicHunter (Fig. 2A). In addition, 68 of 101 sSNVs that were detected by RNA-MosaicHunter but not MuTect showed variant-supporting reads with >2% VAF in the exome-seq data, suggesting that they were true clonal somatic variants omitted by MuTect (Fig. 2A). Among 984 MuTect-called exonic variants with at least 10 RNA-seq reads, RNA-MosaicHunter successfully recaptured 525 of them. The sSNVs missed by RNA-MosaicHunter generally had poor coverage or low VAF in RNA-seq data, likely due to low expression levels or allele-specific expression, leading to underrepresentation of the variant allele in the tumor samples [27]. Overall, RNA-MosaicHunter achieved 53.4% sensitivity (Fig. 2B) and 94.7% precision (Fig. 2C) in identifying sSNVs from these cancer datasets. We further benchmarked RNA-MosaicHunter by selectively disabling individual empirical error filters or by removing all filters. Although these filters reduced sensitivity, they markedly improved precision in somatic variant detection—a crucial feature for accurate profiling in non-cancer tissues with low mutation rates (Fig. 2B and C).

Although we implemented a series of filters in RNA-MosaicHunter to specifically remove RNA-editing sites, some A>G editing sites may remain in the somatic variant call list. To address this, RNA-MosaicHunter includes a mode that fully excludes all A>G/T>C candidates. In this mode, RNA-MosaicHunter slightly reduced the sensitivity to 50.6%, while improving the precision to 94.9% (Fig. 2D). In summary, RNA-MosaicHunter demonstrates high precision with satisfactory sensitivity for somatic variant profiling from bulk RNA-seq data in its default mode, while allowing users to easily adjust filters in the configuration file to prioritize either sensitivity or precision.

### Cell-line mixture experiments validate RNA-MosaicHunter’s performance in non-cancer context

**Figure 3.**
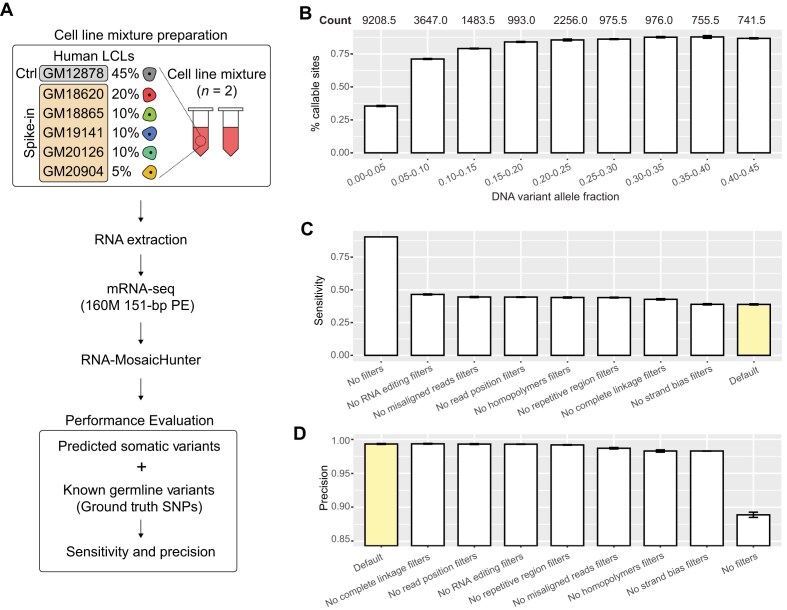
Benchmarking the performance of RNA-MosaicHunter using the cell-line mixture dataset. (**A**) Schematic of the experimental design for preparing cell-line mixture samples. Six human lymphoblastoid cell lines (LCLs) were pooled at different proportions, using WGS-called germline variants to mimic somatic variants with varying variant allele fractions. Ctrl, control; PE, paired-end reads. (**B**) Percentage of sites in the WGS-derived true set with sufficient RNA-seq coverage (>10), variant allele count (>5), and variant allele fraction (>5%) to meet the default RNA-MosaicHunter calling requirements. Average denominator values between the two replicates are shown above each bar. Sensitivity (**C**) and precision (**D**) of RNA-MosaicHunter in default mode and after removing individual empirical error filters or all filters, measured among the callable sites that meet the coverage and fraction thresholds in panel (**B**). Precision was substantially improved by applying the filters, albeit with a corresponding decrease in sensitivity. Error bar, standard deviation.

To evaluate the performance of RNA-MosaicHunter beyond cancer datasets, we conducted additional benchmarking using controlled cell-line mixture experiments. Six human lymphoblastoid cell lines, each previously subjected to whole-genome sequencing for comprehensive genotyping, were pooled at varying proportions. Germline variants present in subsets of the six lines thereby served as surrogates for somatic variants with defined expected allele fractions (Fig. 3A). Two independent biological replicates of the mixtures were prepared and subjected to conventional bulk RNA-seq. This strategy yielded 26 915 simulated somatic variants in RNA-seq-covered regions, including 21 037 with expected allele fractions ranging from 0% to 45% (Fig. 3B), which is the typical range observed for real-world somatic variants. As expected, the proportion of variants supported by sufficient mutant reads for confident calling increased from 35.5% at allele fractions between 0% and 5% to >85% when the allele fractions were over 20% (Fig. 3B).

We next applied RNA-MosaicHunter to the two RNA-seq datasets independently and assessed sensitivity and precision at sites with sufficient mutant-supporting reads for somatic variant calling (Supplementary Table S3). With default parameters, RNA-MosaicHunter achieved an overall sensitivity of 38.9% and precision of 99.3% across all simulated somatic variants (Fig. 3C and D). Consistent with the TCGA benchmarking results, removal of individual empirical error filters slightly increased sensitivity while decreasing precision (Fig. 3C and D). Taking advantage of the larger number of simulated variants, we further evaluated the effects of allele fraction and RNA-seq depth on performance. As shown in Supplementary Fig. S1, RNA-MosaicHunter maintained consistent performance across both parameters, highlighting its robustness. Notably, the precision estimates depend on the relative abundance of true somatic variants versus artifacts. Although the number of artifacts is expected to remain relatively stable across RNA-seq datasets, the number of true somatic variants may be substantially lower in non-cancer samples compared with benchmarking datasets. Therefore, we recommend retaining all error filters to maximize accuracy in such applications.

### RNA-MosaicHunter reveals somatic variant patterns across normal tissue types

**Figure 4.**
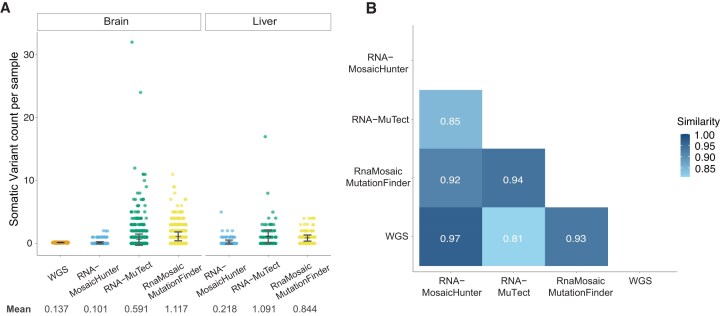
Benchmarking RNA-MosaicHunter to other methods on RNA-seq from GTEx. (**A**) Comparison of the number of somatic sSNVs detected from deep WGS [55] and GTEx RNA-seq by three calling pipelines, RNA-MosaicHunter, RNA-MuTect [32], and RnaMosaicMutationFinder [33]. RNA-MosaicHunter detected fewer variants than other tools in the brain and was comparable to the number of variants that overlapped in the RNA-seq-powered region reported by WGS. Error bar, standard deviation. (**B**) Cosine similarity of brain variant spectrum between WGS-detected variants and three RNA-seq sSNV calling pipelines. RNA-MosaicHunter showed the highest similarity with WGS and outperformed other tools.

Next, we applied RNA-MosaicHunter to identify somatic variants in normal tissue samples from the GTEx dataset. We selected three tissue types—brain, liver, and whole blood—as representatives of the three embryonic germ layers. To further benchmark RNA-MosaicHunter, we compared its results with the somatic variant lists for the same GTEx tissue types called by two previous RNA-based methods: RNA-MuTect [32] and RnaMosaicMutationFinder [33]. Of the three methods, RNA-MosaicHunter reported the closest clonal sSNV burden (0.101 per sample) to the gold-standard burden estimated by deep WGS (0.137 per sample, within RNA-seq powered regions) [55] in brain samples (Fig. 4A); in contrast, RNA-MuTect and RnaMosaicMutationFinder reported 4.3 and 8.2 times higher burdens than the gold standard (Fig. 4A), suggesting that their call lists likely contain many false positives. RNA-MosaicHunter also reported the lowest sSNV burden in liver samples (0.218 per sample) among the three methods (Fig. 4A), in line with previous findings that liver cells accumulate sSNVs two to three times faster than brain cells [66, 67]. We further compared the mutation spectrum of brain sSNVs identified by each of the three RNA-based methods, and again RNA-MosaicHunter achieved the highest cosine similarity to the WGS-based gold standard (Fig. 4B). These results suggest that RNA-MosaicHunter outperforms other tools in accurately identifying somatic variants from real-world non-cancer RNA-seq datasets.

**Figure 5.**
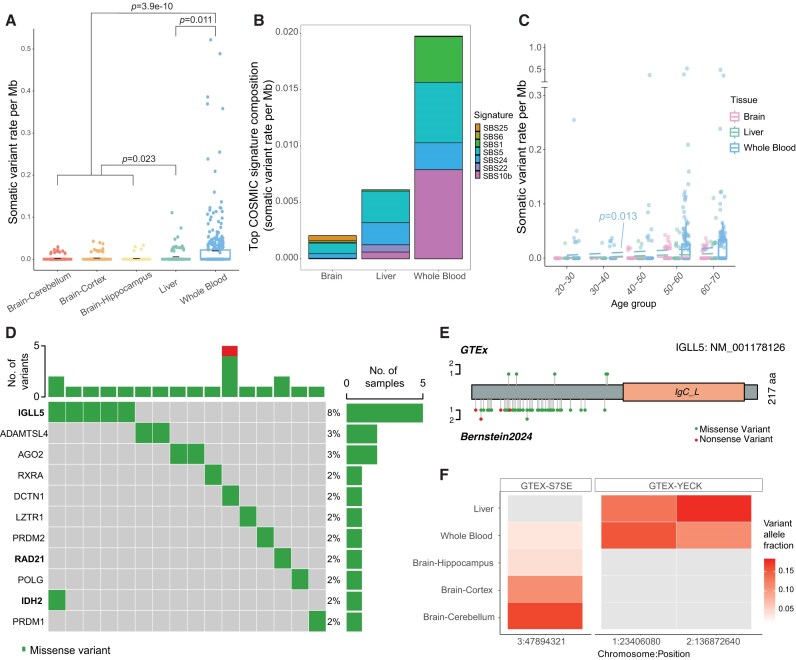
Somatic variant patterns revealed by RNA -MosaicHunter from normal brain, liver, and whole blood samples of GTEx. (**A**) Somatic variant rate per Mb in the brain (cerebellum, cortex, hippocampus), liver, and whole blood. Blood exhibited a significantly higher variant burden than other tissues, followed by the liver and brain. *P*-values were calculated with the Wilcoxon rank-sum exact test with Benjamini–Hochberg correction. (**B**) Contribution of major COSMIC signatures for somatic variants identified in these tissues. Three signatures with minimal contributions are not shown. Somatic variant rate was uniformly corrected by trinucleotide composition in RNA-seq. SBS5 contributed to a similar level across three tissue types, while SBS1 was observed as a dominant feature in sSNVs detected from the blood. (**C**) sSNV rate distribution across different age groups. The variant burden significantly increased with age in blood (*P* = .013, linear regression), but not in other tissues. (**D**) Gene-level distribution of somatic variants identified from the blood. Three known CHIP genes, *IGLL5, RAD21*, and *IDH2*, were highlighted in bold. Only genes with variants captured in multiple samples or the Cancer Gene Census were plotted. (**E**) Distributions of blood somatic variants in *IGLL5* between our study on GTEx RNA-seq and a previous DNA-seq-based CHIP study [70]. Somatic variants in IGLL5 are enriched in the N-terminus. (**F**) Variant allele fraction for shared sSNV across different tissues of the same donors. One sSNV was found to be present in all sampled tissues, with higher VAFs in the brain cortex and cerebellum. Two other variants demonstrated a shared presence between the liver and blood within an individual. The red gradient highlighted the variant allele fraction, while the gray color represented no sample available for the corresponding tissue type. (A, C) Boxplots show the median and the first and third quartiles, while whiskers denote 1.5× interquartile range (IQR) from hinges.

Using RNA-MosaicHunter, we investigated the clonal somatic variant patterns in brain, liver, and whole blood samples from non-cancer GTEx individuals. In total, we identified 274 sSNVs from 827 RNA-seq samples (Supplementary Fig. S2 and Supplementary Tables S4 and S5). After normalizing by the genomic size of RNA-seq-powered region in each sample, we observed that the blood had the highest sSNV burden, followed by the liver and then the brain (Fig. 5A; *P* < .05, Wilcoxon rank-sum test with Benjamini–Hochberg correction), consistent with previous findings that proliferating cells accumulate somatic variants faster than non-proliferating cells like neurons [12]. We further confirmed that the observed rate difference could not be explained by variations in detection sensitivity, as the VAF distribution across all brain regions and tissues showed no significant differences (Supplementary Fig. S3A and B). Our mutational signatures of somatic variants revealed a comparable level of SBS5 contribution across brain, liver, and blood samples (Fig. 5B), as SBS5 is known as an age-related signature that accumulates universally in all cell types [68]. In contrast, the other age-related signature, SBS1, which is closely associated with cell proliferation and consists of C>T variants at CpG sites [69], was primarily observed in blood samples (Fig. 5B and Supplementary Fig. S3C).

When we grouped the GTEx samples by age, we found a significant association between age and sSNV burden only in the blood samples (Fig. 5C; *P* = .013, linear regression). This result aligns with previous findings that clonal somatic variants accumulate with age in blood cells, linked to the clonal expansion of blood cells driven by somatic variants associated with CHIP [70]. Indeed, we found that several cancer-related genes were recurrently hit by somatic variants in multiple non-cancer blood samples (Fig. 5D), including three previously reported CHIP genes: *IGLL5, RAD21*, and *IDH2* [57, 58, 70]. Notably, *IGLL5*, which encodes the immunoglobulin lambda-like polypeptide 5, a protein involved in memory B cell expansion [71] and ​​lymphoid neoplasms reported by COSMIC [72], exhibited blood somatic variants across several individuals; all of these variants were enriched in the N-terminus of the protein (Fig. 5E), consistent with findings from a prior CHIP study based on DNA-seq data [70].

We further investigated whether somatic variants could be shared across multiple tissue types within the same individual and identified three such variants. One of the variants was shared across the brain cortex, cerebellum, hippocampus, and blood, though its VAFs were significantly lower in the latter two tissues, preventing detection by RNA-MosaicHunter (Fig. 5F). Two variants from another individual were shared between the liver and blood; however, since brain samples were unavailable, we cannot determine whether these variants were also present in brain tissues (Fig. 5F).

Overall, our analysis of GTEx data revealed a dynamic accumulation of clonal somatic variants in normal blood samples with aging, reflecting the characteristics of cell proliferation and CHIP. In contrast, brain and liver samples exhibited a much lower burden of clonal somatic variants, likely due to lower cell turnover rates and more spatially-restricted clonal architectures in these tissues.

### Higher burden of clonal somatic variants in AD cortex

**Figure 6.**
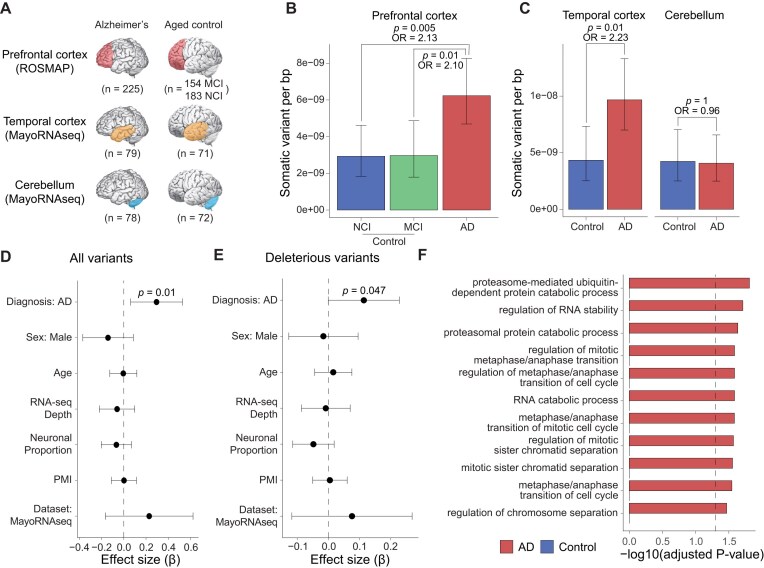
RNA-MosaicHunter reveals an elevated burden of somatic variants in the cerebral cortex of AD patients. (**A**) Transcriptome-wide screen of sSNVs among 862 RNA-seq datasets of AD and control brain samples from ROSMAP [59] and MayoRNAseq [60] datasets. Somatic variants were called by RNA-MosaicHunter. MCI, mild cognitive impairment; NCI, no cognitive impairment. (**B, C**) Greater variant burden in cerebral cortex samples of AD patients when compared to matched controls. A significant two-fold increase of sSNV density in AD prefrontal cortex and temporal cortex was consistently found in both ROSMAP (**B**) and MayoRNAseq (**C**) cohorts. The burden increase was not observed in the AD cerebellum. CI, cognitive impairment. (**D**) Linear regression modeling confirms that the sSNV increase in AD brains remains significant after controlling for potential covariates. PMI, post-mortem interval. (**E**) AD brains had significantly more deleterious sSNVs than controls (*P* = .047, linear regression) after controlling for potential confounding factors. (**F**) GO terms enriched for AD sSNVs. Genes regulating cell cycle and proliferation are specifically enriched for AD but not control sSNVs. (**B**–**E**) Error bar, 95% CI.

Somatic variants in the brain have been recently associated with neurodegenerative diseases, including AD [67, 73, 74]. Here, we applied RNA-MosaicHunter to 862 brain RNA-seq datasets generated by two large-scale AD cohorts, ROSMAP [59] and MayoRNAseq [60] (Fig. 6A and Supplementary Table S6). In each dataset, the AD and control samples were matched for sex, age (Supplementary Fig. S4A), post-mortem interval, and sequencing depth (Supplementary Fig. S4B). Across these samples, RNA-MosaicHunter identified a total of 178 sSNVs in AD and control brains (Supplementary Table S7).

From the ROSMAP cohorts, AD PFC samples exhibited a significantly higher burden of somatic variants compared to controls with no or only mild cognitive impairment (Fig. 6B; *P* < .01, two-tailed proportion test; OR = 2.1). This finding was further validated in a second, independent RNA-seq dataset from MayoRNAseq, where AD temporal cortex samples showed a consistent increase of sSNV burden compared to neurotypical controls (Fig. 6C; *P* = .01, two-tailed proportion test; OR = 2.2), with a remarkably similar odds ratio to that seen in the ROSMAP PFC samples. Interestingly, the disease-specific enrichment of sSNVs was observed only in the temporal cortex and not in the cerebellum (Fig. 6C; *P* = 1, two-tailed proportion test), a brain region not severely affected in AD [75]. The observed greater sSNV burden in AD remained significant after controlling for potential confounding factors, including sex, age, RNA-seq coverage, post-mortem interval, neuronal proportion, and batch effects (Fig. 6D and Supplementary Fig. S4A and B; *P* = .01, linear regression). This enrichment persisted even when only the subset of sSNVs predicted to have a deleterious impact on protein function was considered (Fig. 6E and Supplementary Fig. S4C; *P* = .047, linear regression).

Next, we compared the composition of variant types for sSNVs identified from AD and control brain samples. A previous single-neuron WGS study [67] reported an increased burden of sSNVs in AD brains, driven primarily by a mutational signature dominated by C>A/G>T variants, which likely reflects elevated oxidative stress during AD pathogenesis. Consistently, we observed a similar trend in our RNA-MosaicHunter results, with sSNVs from AD samples exhibiting a higher proportion of C>A/G>T variants than those from controls (Supplementary Fig. S4D; 22.4% in AD versus 17.2% in control). We further examined the distribution of these somatic variants across different gene functions. Using GO annotation, we observed that sSNVs found in AD brains were significantly enriched in genes related to ubiquitin-dependent proteolysis (Fig. 6F), which has been reported to be associated with AD [76]. Moreover, there was an enrichment of genes that regulate cell cycle and proliferation (adjusted *P* < .05, hypergeometric test), which was not found in sSNVs identified in control brains (Fig. 6F).

To summarize, by applying RNA-MosaicHunter to two distinct AD cohorts, we consistently observed approximately a two-fold increase in clonal somatic variants in AD brain cortex samples compared to matched controls, underscoring the potential role of brain somatic variants in increasing AD risk. These AD somatic variants were specifically enriched in genes that regulate cell cycle and proliferation, aligning with previous reports that such proliferation-related somatic variants, particularly in microglia, may contribute to the pathogenesis of neurodegeneration [11, 77–79].

## Discussion

Detecting somatic variants from RNA-seq data has been a demanding challenge, with existing tools often lacking reproducibility and generalizability, particularly for non-cancer samples. We introduce RNA-MosaicHunter, a novel tool designed to accurately identify clonal somatic variants from bulk RNA-seq data in both cancerous and non-cancerous tissues. Through benchmarking on TCGA cancer, cell-line mixture, and GTEx normal tissue datasets, we demonstrated that RNA-MosaicHunter generally outperforms previous tools, particularly with its high precision, leading to a more accurate estimation of somatic variant burden in normal tissues. We further applied RNA-MosaicHunter to profile somatic variants in AD and control brain samples from the ROSMAP and MayoRNAseq datasets and observed a significant enrichment of somatic variants in the cortex of AD brains, highlighting the potential contribution of somatic variants to AD pathogenesis.

Excluding RNA-editing sites is a critical step in somatic variant calling from RNA-seq data. In addition to filtering out variants listed in existing RNA editing databases, we provided a filter that specifically removes RNA-editing sites by considering both mutation type and gene transcription direction. After applying these RNA-editing filters, we achieved a high concordance in the burden and spectrum of somatic variants compared to DNA-based methods (Fig. 4). However, it is important to note that these filters may inadvertently discard real A>G somatic variants if they share characteristics with RNA editing. Thus, RNA-MosaicHunter also allows users to disable RNA-editing filters or flag, rather than remove, these filtered sites, providing greater flexibility for specific applications.

Analysis of the GTEx dataset revealed that the brain exhibits the lowest somatic variant burden, followed by the liver and the blood. Since neurons are the predominant cell type in the brain and are generally post-mitotic after birth, somatic variants accumulated in neurons during aging cannot form large clones and thus remain undetectable by bulk sequencing methods. This aligns with our findings that show no age-related accumulation trend in GTEx brain samples (Fig. 5C). On the other hand, liver and blood cells continue to proliferate in adult humans: hepatocytes have an average age of 2.7–2.9 years in adult humans, with a 17%–19% birth rate each year [80], whereas most blood cells display a significantly faster turnover rate compared to liver cells, with a turnover rate varying from a few hours to a few months [81, 82]. Previous single-cell sequencing studies reported that liver cells may accumulate somatic variants faster than blood cells [66, 83], though our analyses showed that the blood exhibits a stronger age-dependent accumulation of clonal somatic variants than the liver. This inconsistency could be explained by differences in clonal architecture between the two tissues: blood cells may more readily expand into larger clones that dominate the blood cell pool [84], allowing these variant-carrying clones to be captured in bulk RNA-seq data, whereas liver clones are more focal and spatially restricted [85], resulting in a lower likelihood of detection by conventional bulk tissue sequencing used in GTEx, unless clones are carefully microdissected.

Our analysis of two AD datasets consistently revealed a higher burden of somatic variants in the prefrontal and temporal cortex of AD patients compared to age-matched controls; however, cerebellum samples from AD patients in the same cohort showed a similar burden to both control cerebellum and cortex samples (Fig. 6C). Unlike the cerebral cortex, the cerebellum is relatively intact in the early stages of AD and is significantly less affected by the disease’s pathological processes, with much lower accumulation of amyloid-beta and tau pathology [75, 86]. Together with the previous single-cell WGS study showing elevated somatic variant burdens in neurons of the PFC and hippocampus that are associated with increased oxidative stress in AD [67], our findings suggest that somatic variants in the cerebral cortex may play a role during AD pathogenesis.

Currently, RNA-MosaicHunter employs empirical filters to mitigate artifacts attributable to various biological processes and sequencing errors. Moving forward, the integration of deep learning models promises to enhance the discrimination of true somatic variants from false positives, particularly as large-scale training datasets for somatic variants become available. Furthermore, we plan to expand the capacity of our tool to analyze single-cell RNA-seq data, enabling a more detailed exploration of somatic variants across various cell types in human tissues. In summary, RNA-MosaicHunter opens the possibility for somatic variant profiling in existing large-scale RNA-seq datasets, enabling a better understanding of the characteristics and contributions of somatic variants in human tissue and pathology.

## Supplementary Material

gkaf1450_Supplemental_Files

## Data Availability

RNA-MosaicHunter is publicly available at https://doi.org/10.6084/m9.figshare.28314569.v4 and https://github.com/AugustHuang/RNA-MosaicHunter. The RNA-seq data of the cell-line mixture experiments have been deposited with the Sequence Read Archive under accession number PRJNA1335185.

## Notes

Present Address: Division of Biology & Biomedical Sciences, Washington University in St. Louis, St Louis, MO 63108, United States
