# Supplementary material for: Accurate detection of somatic single-nucleotide variants from bulk RNA-seq data using RNA-MosaicHunter
Source: Nucleic Acids Res. 2026 Jan 8;54(1):gkaf1450. doi: 10.1093/nar/gkaf1450 (PMC12781890; doi:10.1093/nar/gkaf1450)
Supplement: gkaf1450_Supplemental_Files [file gkaf1450_supplemental_files.zip › Manuscript_RNA-MosaicHunter_Supplementary_data.pdf]

Supplementary Data

Accurate detection of somatic single-nucleotide variants from bulk RNA-seq data using RNA-MosaicHunter

August Yue Huang[1,2,3,*,†], Yuchen Cheng[1,4,†], Jayoung Ku[1,2,3], Boxun Zhao[1,2,3], Junseok Park[1,2], Dachan Kim[1,2,5], Jaejoon Choi[1,2,3], Eunjung Alice Lee[1,2,3,*]

[1] Division of Genetics and Genomics and Manton Center for Orphan Disease Research, Boston Children's Hospital, Boston, MA, 02115, USA.
[2] Department of Pediatrics, Harvard Medical School, Boston, MA, 02115, USA.
[3] Broad Institute of MIT and Harvard, Cambridge, 02142, MA, USA
[4] Department of Biomedical Informatics, Harvard Medical School, 02115, MA, USA
[5] Department of Otorhinolaryngology, Severance Hospital, Yonsei University Health System, Yonsei University College of Medicine, Seoul, 03722, South Korea.

*To whom correspondence should be addressed. Email: ealice.lee@childrens.harvard.edu, yue.huang@childrens.harvard.edu

†The authors wish it to be known that, in their opinion, the first two authors should be regarded as joint First Authors

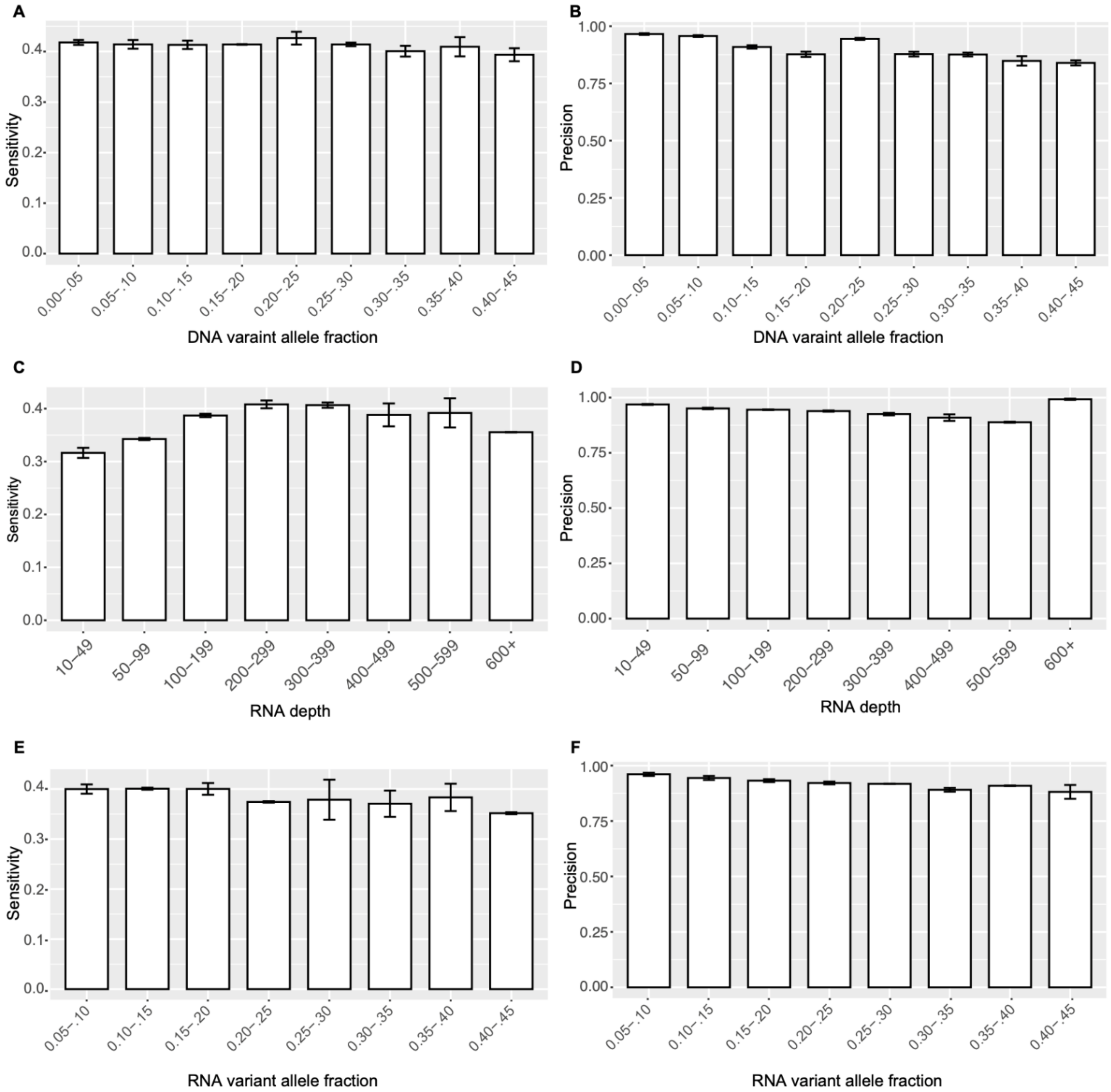

Supplementary Figure S1. Performance of RNA-MosaicHunter on the cell-line mixture dataset. Sensitivity (**A, C, E**) and precision (**B, D, F**) of RNA-MosaicHunter in default mode on the cell-line mixture dataset, stratified by DNA VAF (**A-B**), RNA sequencing depth (**C-D**), or RNA VAF (**E-F**). Calculations were performed using the same strategy as in **Figure 3C-D**.

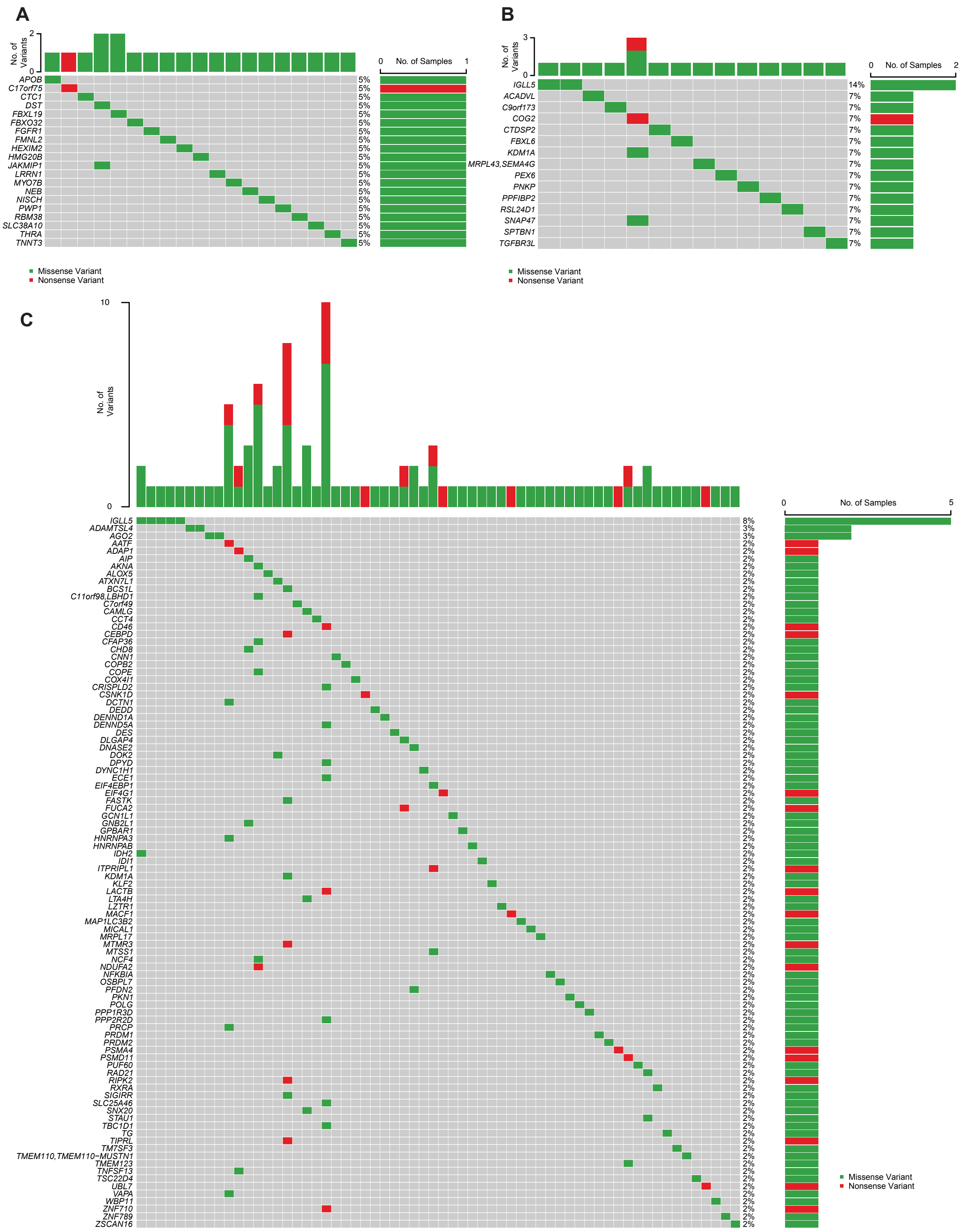

Supplementary Figure S2. Full list of protein-altering somatic variants identified by RNA-MosaicHunter from GTEx brain (A), liver (B), and whole blood (C) tissues. Missense and nonsense variants are shown in green and red, respectively.

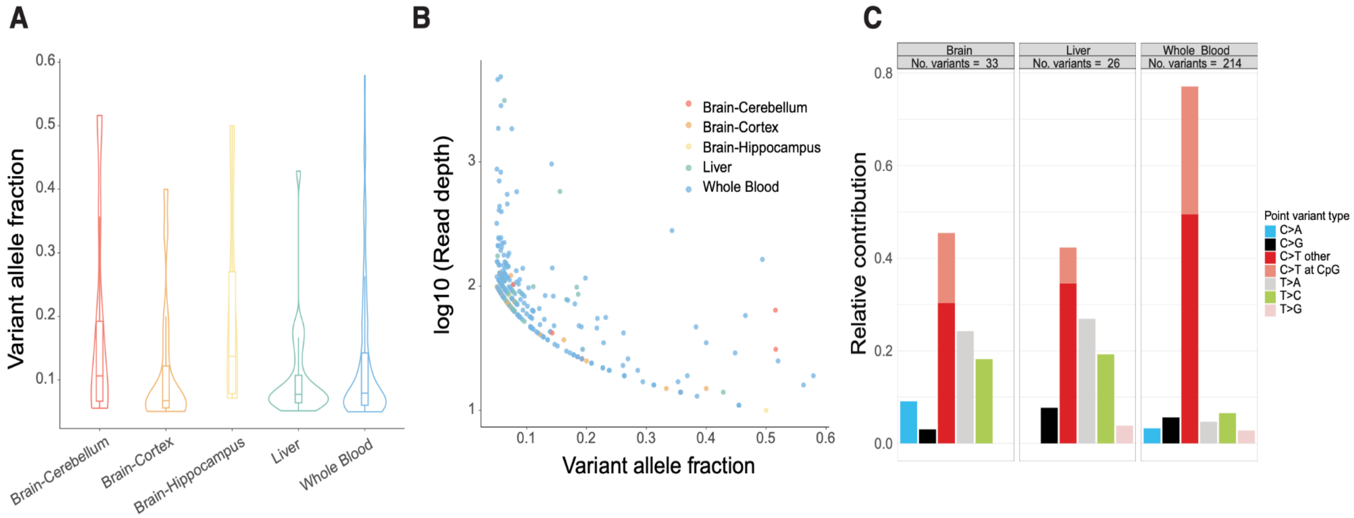

Supplementary Figure S3. Variant allele fraction and variant type of somatic variants detected from GTEx dataset. (**A**) Variant allele fraction of sSNVs detected from the brain cerebellum, cortex, hippocampus, liver, and whole blood. There is no statistical significance of VAF among groups (Wilcoxon rank sum exact test with Benjamini-Hochberg correction, N.S.). Boxplots show the median and the first and third quartiles, with whiskers denoting 1.5 * IQR from hinges. (**B**) Correlation of variant allele fraction and read depth. Deeper read depth had a higher potential for detecting somatic variants with lower variant allele fraction. (**C**) Variant types identified in brain, liver, and whole blood. Blood had a higher proportion of C>T variants, especially at CpG sites, than other tissues.

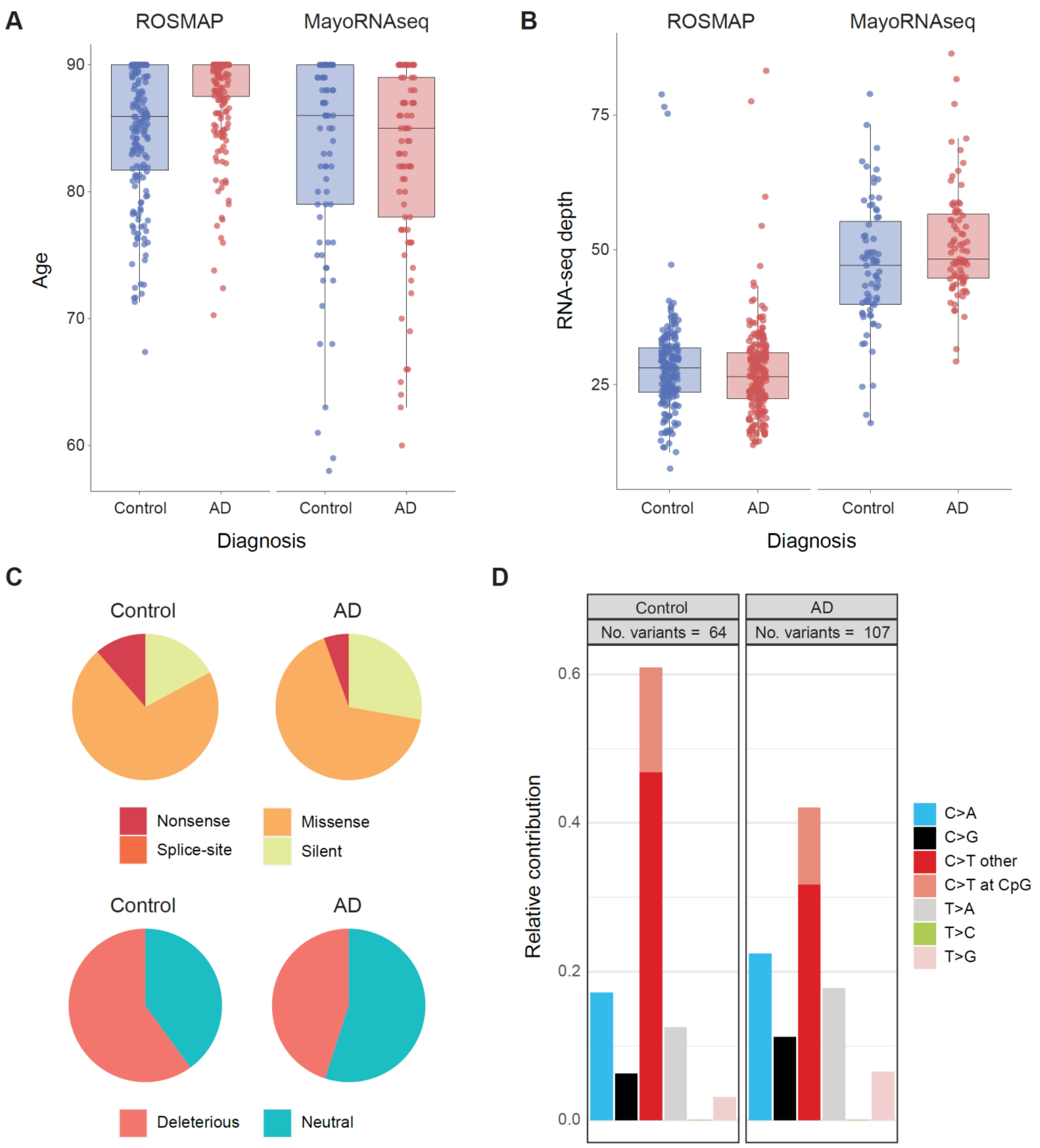

Supplementary Figure S4. RNA-MosaicHunter reveals somatic variant patterns in AD brain samples. (**A**) Age distribution of AD and control samples collected from ROSMAP and MayoRNAseq. (**B**) Similar sequencing depth between the AD and control brain samples in each AD cohort. The overall higher depth in MayoRNAseq may explain the higher base-line variant burden in control brain samples than ROSMAP. Boxplots show the median and the first and third quartiles, with whiskers denoting 1.5 * IQR from hinges. (**C**) Genic annotation and functional impact prediction of sSNVs identified from AD and control brain samples. (**D**) Variant type of sSNVs detected from AD and control brain samples.

Supplementary tables

Supplementary Table S1: Sample list and metadata for TCGA esophageal carcinoma samples

Supplementary_Table_S1_TCGA_sample_list.xlsx

Supplementary Table S2: Mutation list for TCGA esophageal carcinoma samples

Supplementary_Table_S2_TCGA_mutation_list.xlsx

Supplementary Table S3: Mutation list for cell-line mixture samples

Supplementary_Table_S3_cell_line_mixture_mutation_list.xlsx

Supplementary Table S4: Sample list and metadata for GTEx samples

Supplementary_Table_S4_GTEx_sample_list.xlsx

Supplementary Table S5: Mutation list for GTEx samples

Supplementary_Table_S5_GTEx_mutation_list.xlsx

Supplementary Table S6: Sample list and metadata for AD samples

Supplementary_Table_S6_AD_sample_list.xlsx

Supplementary Table S7: Mutation list for AD samples

Supplementary_Table_S7_AD_mutation_list.xlsx
